# Nitrogen Source Governs Community Carbon Metabolism in a Model Hypersaline Benthic Phototrophic Biofilm

**DOI:** 10.1128/mSystems.00260-20

**Published:** 2020-06-09

**Authors:** Christopher R. Anderton, Jennifer M. Mobberley, Jessica K. Cole, Jamie R. Nunez, Robert Starke, Amy A. Boaro, Yasemin Yesiltepe, Beau R. Morton, Alexandra B. Cory, Hayley C. Cardamone, Kirsten S. Hofmockel, Mary S. Lipton, James J. Moran, Ryan S. Renslow, James K. Fredrickson, Stephen R. Lindemann

**Affiliations:** a Environmental Molecular Sciences Laboratory, Pacific Northwest National Laboratory, Richland, Washington, USA; b Biological Sciences Division, Pacific Northwest National Laboratory, Richland, Washington, USA; c National Security Directorate, Pacific Northwest National Laboratory, Richland, Washington, USA; d Gene and Linda Voiland School of Chemical Engineering and Bioengineering, Washington State University, Pullman, Washington, USA; e Whistler Center for Carbohydrate Research, Department of Food Science, Purdue University, West Lafayette, Indiana, USA; f Department of Nutrition Science, Purdue University, West Lafayette, Indiana, USA; University of British Columbia

**Keywords:** carbon cycling, cyanobacteria, mass spectrometry, nitrogen cycling, stable isotopes

## Abstract

Increasing anthropogenic inputs of fixed nitrogen are leading to greater eutrophication of aquatic environments, but it is unclear how this impacts the flux and fate of carbon in lacustrine and riverine systems. Here, we present evidence that the form of nitrogen governs the partitioning of carbon among members in a genome-sequenced, model phototrophic biofilm of 20 members. Consumption of NO_3_^−^ as the sole nitrogen source unexpectedly resulted in more rapid transfer of carbon to heterotrophs than when NH_4_^+^ was also provided, suggesting alterations in the form of carbon exchanged. The form of nitrogen dramatically impacted net community nitrogen, but not carbon, uptake rates. Furthermore, this alteration in nitrogen form caused very large but focused alterations to community structure, strongly impacting the abundance of only two species within the biofilm and modestly impacting a third member species. Our data suggest that nitrogen metabolism may coordinate coupled carbon-nitrogen biogeochemical cycling in benthic biofilms and, potentially, in phototroph-heterotroph consortia more broadly. It further indicates that the form of nitrogen inputs may significantly impact the contribution of these communities to carbon partitioning across the terrestrial-aquatic interface.

**IMPORTANCE** Anthropogenic inputs of nitrogen into aquatic ecosystems, and especially those of agricultural origin, involve a mix of chemical species. Although it is well-known in general that nitrogen eutrophication markedly influences the metabolism of aquatic phototrophic communities, relatively little is known regarding whether the specific chemical form of nitrogen inputs matter. Our data suggest that the nitrogen form alters the rate of nitrogen uptake significantly, whereas corresponding alterations in carbon uptake were minor. However, differences imposed by uptake of divergent nitrogen forms may result in alterations among phototroph-heterotroph interactions that rewire community metabolism. Furthermore, our data hint that availability of other nutrients (i.e., iron) might mediate the linkage between carbon and nitrogen cycling in these communities. Taken together, our data suggest that different nitrogen forms should be examined for divergent impacts on phototrophic communities in fluvial systems and that these anthropogenic nitrogen inputs may significantly differ in their ultimate biogeochemical impacts.

## Abstract (precis)

Anthropogenic inputs of nitrogen into aquatic ecosystems, and especially those of agricultural origin, involve a mix of chemical species. Although it is well-known in general that nitrogen eutrophication markedly influences the metabolism of aquatic phototrophic communities, relatively little is known regarding whether the specific chemical form of nitrogen inputs matter. Our data suggest that the nitrogen form alters the rate of nitrogen uptake significantly, whereas corresponding alterations in carbon uptake were minor. However, differences imposed by uptake of divergent nitrogen forms may result in alterations among phototroph-heterotroph interactions that rewire community metabolism. Furthermore, our data hint that availability of other nutrients (i.e., iron) might mediate the linkage between carbon and nitrogen cycling in these communities. Taken together, our data suggest that different nitrogen forms should be examined for divergent impacts on phototrophic communities in fluvial systems and that these anthropogenic nitrogen inputs may significantly differ in their ultimate biogeochemical impacts.

## INTRODUCTION

Linkages between biogeochemical carbon, nitrogen, and other micronutrient cycling exert significant influences on global geochemical processes (1, 2). Many studies exploring nitrogen’s impact on carbon cycling in terrestrial systems, for example, have focused on the influences of fertilizer amendments (3, 4) or nitrogen fixation in aqueous environments (5, 6). However, little is known about whether and how the molecular state in which these elemental resources are taken up by microbial communities affects the biotic processing and cycling of the other elements. This limits our understanding of interactions among elemental cycles and, in turn, our ability to predict the outcomes of alterations in microbially mediated elemental cycling. One example is how substantial projected increases in anthropogenic N inputs (e.g., from agriculture, which are typically composed of urea, NO_3_^−^, NH_4_^+^, or mixes thereof) (7) into marine, lacustrine, and fluvial systems (8, 9) will affect the flux and fate of C within these environments.

Phototrophic consortia, like those we have extensively characterized from Hot Lake, WA (10–16), are ubiquitous in aquatic environments worldwide. Therefore, elucidating the carbon, nitrogen, and other micronutrient metabolic linkages within these microbial communities can notably advance our knowledge of the mechanisms that govern elemental flow and inform models describing how these communities behave on a global scale. Here, we explored how C metabolism was affected by the available N form within the unicyanobacterial consortium UCC-O (13), a stable, model multispecies biofilm-forming culture that was isolated from the phototrophic microbial mat of Hot Lake, WA (11). Notably, this phototrophic biofilm community’s reproducible assembly across experiments has allowed resource dynamics to be evaluated across multiple independent successions (10, 13). Finally, the metagenome of UCC-O, which has 1 autotrophic member (cyanobacterium *Phormidium* sp. strain OSCR) and 19 associated heterotrophic members has been resolved to the species level (15). This makes it an ideal system to address questions related to coupled biochemical cycling of carbon, nitrogen, and micronutrients because individual species’ responses to different resource conditions can be simultaneously measured for all members.

Here, using a combined stable isotope approach that linked bulk analysis with spatial imaging data afforded us new insights into how the available N form rewired community C metabolism within UCC-O. Our ability to visualize the localization of new C and N allocation, in conjunction with quantitative PCR (qPCR) and proteomic analysis identifying individual member species’ abundance and potential functions, provided deeper details into how community members’ metabolism influenced spatial patterns of C and N exchange in this phototrophic biofilm. These findings imply that the form of anthropogenic N inputs entering fluvial, lacustrine, and coastal systems (and, potentially, bioavailability of the micronutrient iron) may have significant impacts on the mechanisms and rates of C cycling in benthic, phototrophic communities across the terrestrial-aquatic interface globally.

## RESULTS AND DISCUSSION

### Nitrogen source exerts focused effects on community dynamics in the biofilm.

**FIG 1:**
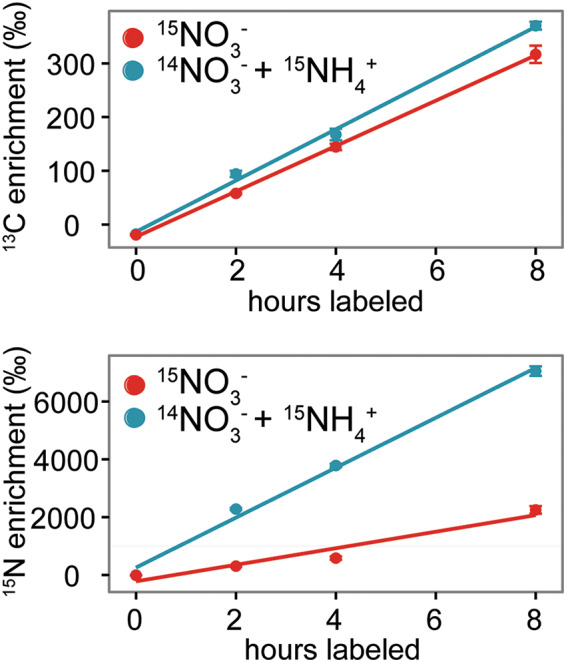
The available nitrogen source affects bulk nitrogen incorporation, but not bulk carbon incorporation into the model phototrophic biofilm. Unicyanobacterial consortium UCC-O was grown in media that contained either NO_3_^−^ with NH_4_^+^ or NO_3_^−^ without NH_4_^+^, and both were supplemented with HCO_3_^−^. The bulk isotope uptake of ^15^N and ^13^C was measured in a 7-day-old biofilm, where the unicyanobacterial consortium was fed H^13^CO_3_^−^ with either ^15^NO_3_^−^ only or with NO_3_^−^ supplemented with ^15^NH_4_^+^ and incubated for 8 h.

Supplementing a reduced N source (NH_4_^+^) into the growth medium of the UCC-O consortium resulted in more rapid N incorporation than an oxidized source (NO_3_^−^) (Fig. 1; see also Fig. S1 in the supplemental material), as observed in other phototrophic systems (2, 17, 18). Many microbial photoautotrophs incorporate NO_3_^−^ only in the absence of available NH_4_^+^. This behavior has been theorized to result from differences in the energetics of NH_4_^+^ versus NO_3_^−^ incorporation, as NO_3_^−^ utilization requires an eight-electron reduction for incorporation into biomolecules (18). Interestingly, however, accessibility to reduced N did not effectively alter net C (HCO_3_^−^) uptake into the community, where we observed only a minor increase in overall ^13^C uptake into the biofilm when the medium was amended with NH_4_^+^. We also found that NH_4_^+^-amended biofilms had nonstatistically different biomass production compared to biofilms amended with only NO_3_^−^ (Fig. S2A), but growth in NH_4_^+^-amended media resulted in diminished chlorophyll *a* and protein content (Fig. S2B and S2C) despite higher rates of overall N incorporation. Based on their genomes, only a few of the heterotrophic species in UCC-O are capable of using NO_3_^−^ as an N source (10). Accordingly, these data suggested that NH_4_^+^ supplementation may have resulted in alleviation of heterotrophic N limitation and, therefore, increased heterotroph growth and decreased the cyanobacterium-to-heterotroph biomass ratio.

**FIG 2:**
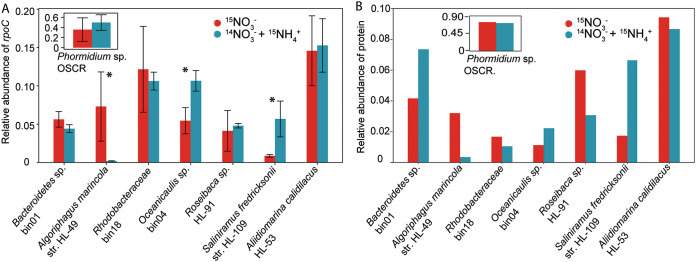
The available nitrogen source affects community dynamics within the model phototrophic biofilm. Unicyanobacterial consortium UCC-O was grown in media that contained either NO_3_^−^ with NH_4_^+^ or NO_3_^−^ without NH_4_^+^, and both media were supplemented with HCO_3_^−^. (A) Comparison of the relative abundance differences in the top eight most-abundant community members as a function of NH_4_^+^ supplementation (the 12 other members account for 1% of the total population combined). The qPCR primer and probe sequence for each of these members can be found in Table S1 in the supplemental material. Significant shifts in HL-49, HL-109, and bin04 population occurred depending on whether the consortium was supplied a reduced form of N (Welch’s independent *t* test, *P* < 0.01; indicated by an asterisk). (B) Bulk proteomic data illustrating the relative number of expressed proteins (by peptide count) per each community member. The total number of proteins being expressed by each member is proportional to the community member’s biomass. str., strain.

Estimating the major species’ abundances based on qPCR of the single-copy *rpoC* gene revealed no significant difference in genome counts for the cyanobacterium *Phormidium* sp. strain OSCR, between medium growth conditions (Fig. 2A, inset). However, we did observe significant shifts in genome counts and peptides originating from key heterotrophic species within the consortial biofilm, which indicated likely shifts in community structure and metabolism. Specifically, qPCR revealed that Algoriphagus marincola HL-49, which possesses no genes involved in nitrate reduction and is thought to be involved in necromass consumption and biomass turnover (10), was highly abundant in biofilms grown in media containing NO_3_^−^ only, but was practically nonexistent in NH_4_^+^-amended biofilms. Conversely, *Saliniramus* (formerly *Salinivirga*) *fredricksonii* HL-109 (10, 12), whose genome also lacked nitrate reduction genes, was much more abundant in NH_4_^+^-amended biofilms. Apart from *Oceanicaulis* sp. strain HLUCCA04 (bin04), which was elevated in abundance in NH_4_^+^-amended media, none of the other major heterotroph abundances (i.e., Aliidiomarina calidilacus HL-53, *Roseibaca* sp. strain HL-91, *Bacteroides* sp. bin01, or *Rhodobacteraceae* sp. bin18) changed significantly as observed by qPCR.

10.1128/mSystems.00260-20.7 TABLE S1 Quantitative PCR primer and probe sequences targeting the *rpoC* gene. Download Table S1, DOCX file, 0.02 MB. Copyright © 2020 Anderton et al. 2020 Anderton et al. This content is distributed under the terms of the Creative Commons Attribution 4.0 International license.

As nucleic acid and protein quantitation methods are known to vary significantly across these organisms (10) and protein is a better estimate of cellular biomass, we also evaluated organismal global protein abundances (Fig. 2B). Peptide abundances displayed some differences, but they confirmed the N-source-responsive behavior. The proteomics revealed opposing changes in abundance between A. marincola HL-49 and S. fredricksonii HL-109 populations. Remarkably, although we had hypothesized that NH_4_^+^ amendment would alleviate heterotrophic N limitations imposed by their inability to assimilate NO_3_^−^ and increase heterotroph abundances broadly, the effects on heterotrophic abundance appeared largely restrained to these two species. Paradoxically, despite the improved access to and energetic favorability of NH_4_^+^ assimilation (17, 18), NH_4_^+^ amendment resulted in only moderate changes in heterotroph abundances. These results suggested that relatively modest alterations in heterotroph abundances were associated with comparatively large changes in overall consortium biofilm phenotype.

Searching for clues to the mechanisms driving this outcome, we analyzed heterotroph protein expression in detail (see Table S3 in the supplemental material). Although in many cases the total number of proteins observed for each member within a single biological replicate was proportional to the community member’s population size under the different conditions as determined by qPCR, *Roseibaca* sp. strain HL-91 produced a much larger relative share of the total protein when NO_3_^−^ was the sole N source. *Bacteroides* sp. bin01 displayed the opposite pattern, with increased protein abundance in NH_4_^+^-amended biofilms. We hypothesized that elevated per-genome *Roseibaca.* sp. HL-91 activity was due in part to its ability to reduce NO_3_^−^ to NO_2_^−^ (10), which might permit a cryptic ability to grow using NO_3_^−^ as an N source, despite predictions that this organism could not perform assimilatory nitrite reduction. Our proteomic results supported this, as we measured nearly fivefold increases in peptide counts related to the respiratory nitrate reductase proteins when NO_3_^−^ was the sole N source (Table S2). It is possible that assimilatory nitrate reduction occurs in interaction with other organisms that can dissimilatorily reduce NO_2_^−^ to NH_4_^+^ (e.g., *Bacteroidetes* sp. bin01). Further, we observed expression of nitrogen regulatory proteins (P-II family) under these conditions and did not detect any peptide counts related to these proteins in biofilms grown in NH_4_^+^-amended media. Increases in *Bacteroidetes* sp. bin01 expression were accompanied by strongly increased expression of pyruvate-ferredoxin oxidoreductases, an enzyme complex involved in carbon metabolism, in NH_4_^+^-amended media. However, the large increases in HL-109 abundance and protein expression and corresponding decreases in strain HL-49 with NH_4_^+^ amendment obscured likely alterations in HL-49 and HL-109 metabolism observable from differences in protein expression. Taken together, these data suggested that NH_4_^+^ supplementation resulted in alterations to proteins involved in heterotrophic nitrogen metabolism, but induced a correspondingly larger number of significant alterations to genes involved in carbon metabolism.

10.1128/mSystems.00260-20.8 TABLE S2 Measured peptide counts related to nitrate utilization of *Roseibaca* sp. HL-91. Download Table S2, DOCX file, 0.01 MB. Copyright © 2020 Anderton et al. 2020 Anderton et al. This content is distributed under the terms of the Creative Commons Attribution 4.0 International license.

10.1128/mSystems.00260-20.9 TABLE S3 Peptide identifications and counts from proteomic analysis. Download Table S3, XLSX file, 0.9 MB. Copyright © 2020 Anderton et al. 2020 Anderton et al. This content is distributed under the terms of the Creative Commons Attribution 4.0 International license.

### Nitrogen form controls allocation of carbon across the biofilm community.

**FIG 3:**
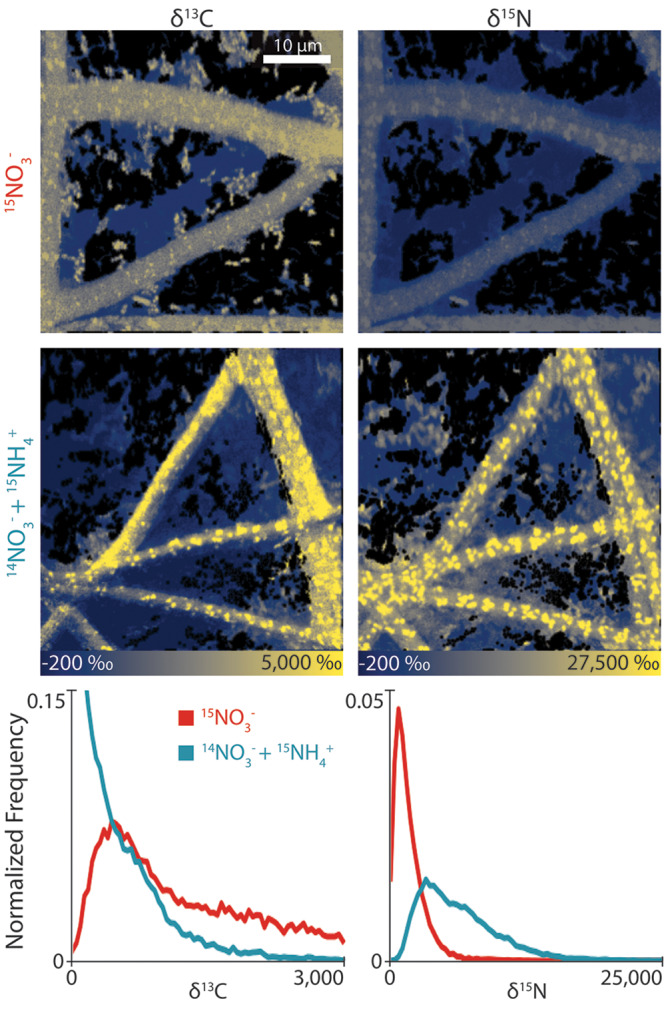
High-lateral-resolution isotopic imaging (256 pixels by 256 pixels, 40 μm by 40 μm) measurements of the biofilms using nanoscale secondary ion mass spectrometry (NanoSIMS) after an 8-h incubation with isotope media. Under the unicyanobacterial consortium growth conditions noted in Fig. 1 and 2, we observed differences in the NanoSIMS images of these biofilms based on ^13^C enrichment (left panels) and ^15^N enrichment (right panels). Using the spatial segmentation methodology developed in our lab for analyzing these images (20), we determined the enrichment of both ^13^C and ^15^N across the entire heterotroph population imaged (histograms in bottom panels). These results quantify the trends observed in the NanoSIMS images themselves. The segmentation images that correspond to this data are in Fig. S6.

To identify actual differences in new C and N allocation under different N conditions, we visualized UCC-O biofilms pulsed with H^13^CO_3_^−^ and either ^15^NO_3_^−^ or ^15^NH_4_^+^ using high spatial resolution nanoscale secondary ion mass spectrometry (NanoSIMS) (19). As expected, these results showed that ^15^NO_3_^−^ uptake localized primarily within the cyanobacterial filaments, with only rare heterotroph cells displaying any notable isotopic enrichment. In contrast, we observed a significant ^15^NH_4_^+^ uptake in the majority of heterotroph cells (Fig. 3 and Fig. S3). Furthermore, cyanobacterial filaments labeled much more strongly with ^15^NH_4_^+^ than with ^15^NO_3_^−^, and appreciably more strongly than neighboring heterotrophs under both conditions. Surprisingly, although we had observed no significant difference in bulk ^13^C uptake into UCC-O biofilms with and without the NH_4_^+^ amendment, NanoSIMS revealed large differences in how ^13^C was partitioned between the two growth conditions after an 8-h incubation with heavy-isotope-enriched media. Where NO_3_^−^ was the sole N source, ^13^C was more readily obtained by heterotrophic cells than when amended with NH_4_^+^. On the other hand, the vast majority of ^13^C was retained within strongly ^15^N-labeled cyanobacterial filaments under NH_4_^+^-amended conditions, with ^13^C incorporation into heterotrophs decreasing with distance from a cyanobacterial filament. Parallel experiments, in which the biofilms were inoculated with labeled media for shorter (2-h) and longer (16-h) periods (Fig. S4), illustrated that domination of ^15^NH_4_^+^ incorporation by *Phormidium* sp. OSCR was not an effect of the labeling period but occurred over all intervals. However, ^15^N enrichment of distal heterotrophs that displayed relatively weak ^13^C incorporation revealed that they were metabolically active but largely consuming carbon fixed before exposure to labeled H^13^CO_3_^−^. Conversely, although the rapid heterotroph incorporation of cyanobacterially fixed ^13^C in NO_3_^−^-only conditions reveals their metabolic activity, they were presumably incorporating nitrogen assimilated prior to exposure to ^15^NO_3_^−^. These data suggest an important role for recycling of necromass to support incorporation of newly fixed C under unamended conditions via N turnover, providing an important community role for *A. marincola* HL-49 which is likely detritivorous (10), which possibly explains its higher abundance under these conditions. Taken together, these observations suggest a strong nitrogen source-dependent difference in linked C and N exchange among members of the consortium.

To provide a more quantitative view of metabolic incorporation across the entire heterotrophic population, we utilized the NanoSIMS image segmentation pipeline we developed previously (20). This permitted us to move from a qualitative view of the metabolic processes within the biofilms (i.e., image visualization by eye) to quantitation of the elemental allocation at the level of each individual cell or pixel. These analyses resolved the C and N flow trends observed in the NanoSIMS images directly on a per-cell (and per-pixel) basis, especially the pronounced shift toward ^13^C incorporation (δ^13^C_ave_ [ave stands for average] = 1,328.39‰ ± 986.23‰) into the heterotroph population in the ^15^NO_3_^−^-only growth conditions (Fig. 3, bottom) and the lack of ^15^N enrichment into the heterotrophic members (δ^15^N_ave_ = 1,814.13‰ ± 1,437.14‰). In NH_4_^+^-amended media, we measured little ^13^C enrichment across the heterotroph population (δ^13^C_ave_ = 121.38‰ ± 26.27‰), but relatively more ^15^N enrichment into these cells (δ^15^N_ave_ = 6,351.97‰ ± 3,549.2‰) than in ^15^NO_3_^−^-only growth conditions. Using the Wilcoxon signed rank test, we found these populations to be significantly different in δ^13^C (*W* = 1.3E6; *P =* 0.000) and δ^15^N (*W* = 6E5; *P =* 0.000). Similar trends were observed in replicate experiments (Fig. S3; Fig. S5 contain associated validation data). Our NanoSIMS image segmentation results also quantitatively supported high autotroph (*Phormidium* sp. OSCR) ^13^C and ^15^N coenrichment compared with heterotrophs grown under all medium conditions. These results showed significantly higher autotroph ^13^C and ^15^N enrichment when grown in NH_4_^+^-amended media than in nonamended media. Finally, these data also suggest that NH_4_^+^ availability permits *Phormidium* sp. OSCR to incorporate more of its fixed C into biomass compared with growth in NO_3_^−^. They further imply that, in the absence of NO_3_^−^, mismatches between the rate of photosynthetic C incorporation and N assimilation may require rapid export of large quantities of fixed C.

### Nitrogen form controls carbon cycling by rewiring cyanobacterial pyruvate metabolism.

**FIG 4:**
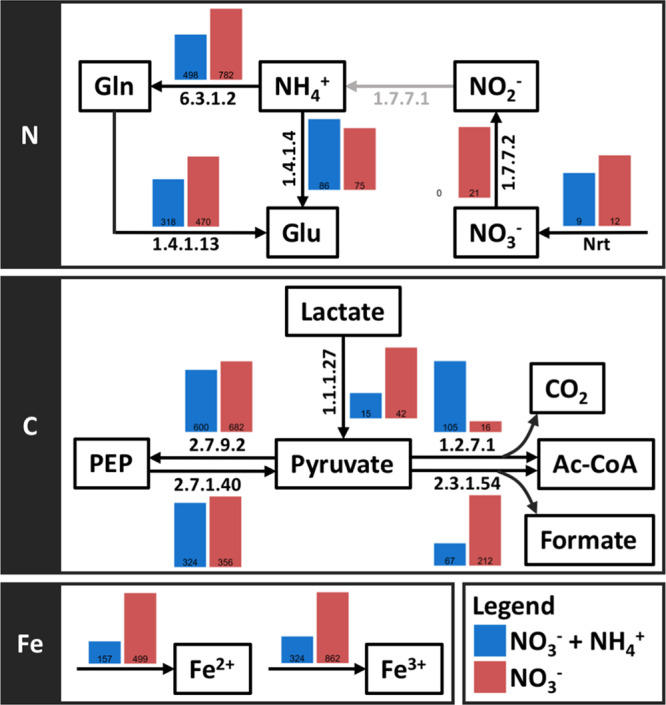
A deeper look into the proteomic data for *Phormidium* sp. strain OSCR. These data suggest changes in C and N metabolism when NH_4_^+^ is added to the media (red), as opposed to when only NO_3_^−^ is available (blue). With respect to nitrogen metabolism, we detected differences in nitrate assimilation via nitrite/nitrate transport transporter (Nrt) and nitrate reductase (EC 1.7.7.2) and in ammonium cycling through glutamine synthetase type 1 (EC 6.3.1.2), glutamate synthase (EC 1.4.13), and glutamate dehydrogenase (EC 1.4.1.4). Proteins in pyruvate metabolism were differentially expressed, and these proteins included pyruvate:ferredoxin oxidoreductase (1.2.7.1), l-lactate dehydrogenase (EC 1.1.1.27), and pyruvate formate-lyase (EC 2.3.1.54). Peptides related to iron acquisition, transport, and use in electron carriers were differentially expressed between treatments.

A deeper proteomic look into the active metabolic pathways within *Phormidium* sp. OSCR revealed N-source-dependent rewiring of central carbon metabolism (Fig. 4). As expected, we observed few peptide counts for proteins involved in nitrate assimilation (i.e., nitrate transport, nitrate reduction, nitrite reduction) when reduced N was provided in NH_4_^+^-amended media, which concurs with bulk isotope incorporation data (Fig. S1, media contained ^15^NO_3_^−^ + ^14^NH_4_^+^). Type I glutamine synthetase and glutamate synthase were upregulated in NO_3_^−^-only conditions, suggesting nitrogen limitation as previously observed at early time points in biofilm formation (10). In contrast, NH_4_^+^ amendment increased expression of glutamate dehydrogenase, which converts glutamate to α-ketoglutarate, suggesting nitrogen-replete conditions favored anapleurotic recycling of amino acids to maintain tricarboxylic acid (TCA) cycle intermediates.

10.1128/mSystems.00260-20.1 FIG S1 Consortial utilization of nitrate is inhibited in the presence of ammonium. The uptake of ^15^N into 7-day-old biofilms fed ^15^NO_3_^−^ only (red), ^15^NO_3_^−^ supplemented with ^14^NH_4_^+^ (purple), ^14^NO_3_^−^ supplemented with ^15^NH_4_^+^ (blue), or the control where ^14^NO_3_^−^ was supplemented with ^14^NH_4_^+^ (green, but barely visible due to a lack of ^15^N label), and incubated for 4 h. Here, we observed that little ^15^N is incorporated into the consortial biomass from ^15^NO_3_^−^ when ^14^NH_4_^+^ is present (purple), and the most ^15^N incorporation into the biomass occurred when ^15^NH_4_^+^ was supplemented into the media (blue). Error bars represent standard deviations. Download FIG S1, DOCX file, 0.02 MB. Copyright © 2020 Anderton et al. 2020 Anderton et al. This content is distributed under the terms of the Creative Commons Attribution 4.0 International license.

10.1128/mSystems.00260-20.2 FIG S2 Nitrogen state availability exerts a minor effect on the bulk properties of consortial biofilms. (A to C) Mean dry weight in 1 ml of homogenized culture (A), chlorophyll *a* amount (1 ml of homogenized culture) (B), and protein mass in 1-ml aliquot (C) in a 7-day-old biofilm, where consortium UCC-O was fed HCO_3_^−^ with either NO_3_^−^ only (red) or with NO_3_^−^ supplemented with NH_4_^+^ (blue). There was no significant change in biomass as a function of nitrogen source. There were significant (Welch’s independent *t* test, *P* < 0.01) decreases in chlorophyll *a* and protein mass in consortia grown in media amended with NH_4_^+^, suggesting the generation of more nonproteinaceous biomass in these biofilms (e.g., exopolysaccharides). Download FIG S2, DOCX file, 0.03 MB. Copyright © 2020 Anderton et al. 2020 Anderton et al. This content is distributed under the terms of the Creative Commons Attribution 4.0 International license.

10.1128/mSystems.00260-20.3 FIG S3 Enrichment trends in biofilm replicates validate observations of C allocation as a function of available N source. Lower-resolution (30 μm by 30 μm, 128 pixels by 128 pixels) images are shown for each condition (^15^NO_3_^−^ only versus NO_3_^−^ supplemented with 15NH_4_^+^, δ^13^C versus δ^15^N). The distribution of δ^13^C and δ^15^N for each image is shown, with transparent lines representing the signature for each of the nine images and the bold line showing the mean signature. Error bars represent the standard deviations at each level of enrichment. Download FIG S3, DOCX file, 2.0 MB. Copyright © 2020 Anderton et al. 2020 Anderton et al. This content is distributed under the terms of the Creative Commons Attribution 4.0 International license.

10.1128/mSystems.00260-20.4 FIG S4 Filamentous cyanobacterium *Phormidium* sp. OSCR is the only autotroph in the community under experimental conditions. NanoSIMS images of UCC-O biofilms fed H^13^CO_3_^−^ with either ^15^NO_3_^−^ only (red) or with NO_3_^−^ supplemented with ^15^NH_4_^+^ (blue) and incubated for 2 or 16 h (left or right columns, respectively). CN^−^ images provide context to the location of biofilm material. δ^15^N images were plotted with bounds of −200 to 37,000‰. All δ^13^C images were plotted with bounds of −200 to 9,000‰. All ^12^C^14^N^−^ images were plotted based on their own minima and maxima, with bounds set to the minimum (image) and 99th percentile (image). Download FIG S4, DOCX file, 1.1 MB. Copyright © 2020 Anderton et al. 2020 Anderton et al. This content is distributed under the terms of the Creative Commons Attribution 4.0 International license.

10.1128/mSystems.00260-20.5 FIG S5 (Left, in color) High- and low-resolution NanoSIMS images show the same enrichment trends for both microbial groups. δ^13^C versus δ^15^N enrichment biplots, comparing autotrophic pixels (cyanobacterial related, green) versus heterotrophic pixels (purple) under conditions of NO_3_^−^ only or with NO_3_^−^ supplemented with NH_4_^+^. Low-resolution images are 128 pixels by 128 pixels, and high-resolution images are 256 pixels by 256 pixels (30 μm by 30 μm or 40 μm by 40 μm, respectively). (Right, in black and white) Partitioning based on cells versus pixels show the same enrichment trends for both microbial groups. δ^13^C versus δ^15^N enrichment biplots, comparing combined autotrophic pixels/cells and heterotrophic pixels/cells under conditions of ^15^NO_3_^−^ only or with NO_3_^−^ supplemented with ^15^NH_4_^+^. Cell-based statistics use full features as a single data point, pixel-based statistics track each pixel within each cell separately. The ellipses represent 95% confidence intervals. This data establishes that we see the same trends whether we measure on a cell-by-cell or pixel-by-pixel basis. The ellipses represent 95% confidence intervals. Download FIG S5, DOCX file, 0.3 MB. Copyright © 2020 Anderton et al. 2020 Anderton et al. This content is distributed under the terms of the Creative Commons Attribution 4.0 International license.

10.1128/mSystems.00260-20.6 FIG S6 Morphological operations and masks can be used to separate autotrophs from heterotrophs. Segmented images related to Fig. 3. Raw ^16^O, ^12^C^14^N, and ^31^P data were used for this operation. First, the log of raw images was calculated and then thresholded to separate high versus low signals in each of these images. Different combinations of the thresholded images were then used to form the autotroph and heterotroph masks. Download FIG S6, DOCX file, 0.3 MB. Copyright © 2020 Anderton et al. 2020 Anderton et al. This content is distributed under the terms of the Creative Commons Attribution 4.0 International license.

With respect to C, NH_4_^+^ amendment markedly altered pyruvate metabolism to acetyl coenzyme A (acetyl-CoA); when available, *Phormidium* sp. OSCR displayed much higher expression of the NrdJ pyruvate-flavodoxin oxidoreductase that oxidizes pyruvate, generating acetyl-CoA and CO_2_. In contrast, when assimilating NO_3_^−^, *Phormidium* sp. OSCR highly expressed l-lactate dehydrogenase, pyruvate formate-lyase, and pyruvate formate-lyase-activating enzyme (Fig. 4). The use of these enzymes suggests an overflow metabolism, as both are nonrespiratory ways to turn over reduced electron carriers that generate surplus organic acids lactate and formate; these can be readily exported and shared with heterotrophs. Alternatively, their use may suggest O_2_ limitation, possibly due to increased heterotrophic biological O_2_ demand; pyruvate formate-lyase employs a radical mechanism that generates toxic reactive oxygen species in the presence of oxygen (21). This result was paradoxical, as the biofilm was maintained under light (and, therefore, presumably O_2_) during the labeling period, but it may reflect either local conditions where rapid heterotroph respiration of organic C fluxes from the cyanobacteria resulted in pockets of hypoxia or internal microcompartments to protect these enzymes from oxygen (22).

Pyruvate formate-lyase is known to be upregulated in iron-limited E. coli chemostatic growth, where it resulted in increased excretion of lactate with more stringent iron limitation (23). We hypothesized that limitation in iron availability may mediate similar pathways in the cyanobacterium, thereby linking C and N cycling in the community. Correspondingly, we observed decreases of ferric and ferrous iron transport proteins by *Phormidium* sp. OSCR when NH_4_^+^ was added to the growth media compared with growth on growth media with NO_3_^−^. Both the cyanobacterial assimilatory nitrate reductase NarB and the nitrite reductase NirA require iron in cofactors—a [3Fe-4S] or [4Fe-4S] cluster in the case of nitrate reductase and a [4Fe-4S] cluster and siroheme in the case of nitrite reductase—and both typically employ ferredoxins [2Fe-2S] as electron carriers (24–26). Furthermore, production of flavodoxin, which can be interchangeable with ferredoxin (27–29), was increased when the biofilm was cultivated in NO_3_^−^ as the sole nitrogen source. This perhaps indicates the necessity to transfer electrons via a non-iron-requiring mechanism. Similar increases in flavodoxin expression have been observed in iron limitation of the cyanobacterium *Synechocystis* sp. strain PCC 6803 (29) and in microbial phototrophs broadly (30, 31). Taken together, these data provide some hints that cyanobacterial iron limitation may, at least in part, mediate the nitrogen source-governed alterations in carbon flux to heterotrophs.

### Conclusion.

Our data broadly support that consumption of NO_3_^−^ as the sole nitrogen source unexpectedly resulted in more rapid transfer of C to heterotrophs than when NH_4_^+^ was provided, hinting at alterations in the form of C exchanged in the UCC-O biofilm. Moreover, the spatial- and species-level resolution data of the element flow revealed that the available N source consumed alters community compositional dynamics, likely via changes in interspecies metabolite exchange among autotrophs and heterotrophs. Notably, these changes markedly affected the population sizes of only two heterotrophic species. Our data suggest that N metabolism may coordinate coupled carbon-nitrogen-iron cycling within these phototrophic biofilm communities, and possibly in other nitrogen- and iron-limited environments such as the open ocean (1). Similar impacts of the N form have been observed with respect to airborne N inputs into natural wetlands (32), engineered wastewater treatment systems (33), and in partitioning of nitrogen among plant roots and associated soil microbes (34), though mechanisms governing these alterations are not well understood.

Here, we present evidence that, in phototrophic consortia, these effects may be mediated by phototroph C responses to the form or rate of N taken up via alterations in phototroph gene expression, which in turn feed back on heterotroph abundances and activities. It should be noted that Hot Lake experiences significant variability in salinity over the course of a seasonal cycle, and the microbial community from which our model community was derived routinely experiences epsomitic hypersalinity (11). It is unknown whether the concentration or identity of the salts involved impact the described biological or abiotic chemical processes. However, if true for other phototrophic communities, the exact mix of available N resources may strongly influence phototroph-heterotroph interactions and, in turn, net C fixation/turnover in aquatic systems. These findings may imply that the form of anthropogenic nitrogen inputs may significantly impact the contribution of autotrophic communities to carbon partitioning across the terrestrial-aquatic interface, and in global carbon fluxes overall. Future studies should investigate whether the form of N inputs in eutrophication indeed influences C dynamics in benthic biofilms.

## MATERIALS AND METHODS

### Biofilm cultivation.

Biofilms were cultivated and harvested as described previously (13). Briefly, cultures were grown under continuous illumination in T75 cell culture flasks (Corning Inc.) for 7 days in 30 ml Hot Lake autotroph 400 (HLA-400) medium (13). The biofilms were scraped with sterile tissue culture scrapers (BD Biosciences) into conical vials and homogenized with sterile, 3-mm glass beads (Thermo Fisher) and pelleted by centrifugation as previously described (13). The pellets were stored at –80°C until DNA extraction or biomass characterization. For NanoSIMS analyses, sterile silicon wafers (5 mm by 5 mm) were implanted in 35-mm tissue culture dishes (Corning Inc.) prior to inoculation. Biofilms colonized these surfaces, which were then carefully removed using forceps for processing, as described below.

### Biofilm biomass characterization.

Dry weight measurements, chlorophyll quantitation, and total protein quantitation were performed using standard protocols on three biological replicates of frozen pellets per measure as described in Supplemental Methods 1 in Text S1 in the supplemental material.

10.1128/mSystems.00260-20.10 TEXT S1 Supplemental methods for biofilm biomass characterization (Supplemental Methods 1), DNA extraction (Supplemental Methods 2), clone library construction for reconstructed genomes (Supplemental Methods 3), and protein extraction, digestion, and LC-MS analysis (Supplemental Methods 4). Download Text S1, DOCX file, 0.02 MB. Copyright © 2020 Anderton et al. 2020 Anderton et al. This content is distributed under the terms of the Creative Commons Attribution 4.0 International license.

### DNA extraction and quantification.

The pellets were washed by adding 1 ml of a solution (pH 8.0) of 500 mM ethylenediaminetetraacetic acid disodium salt dihydrate (EDTA; Sigma-Aldrich) and 550 mM NaCl (Thermo Fisher), 10 min of vortexing at high speed, and centrifugation at 16,000 relative centrifugal force (RCF) at 4°C for 5 min to remove excess Mg^2+^. The supernatant was decanted, and washing was repeated for a total of three washes. The pellets were resuspended in 700 μl of a solution (pH 8.0) of 50 mM Tris(hydroxymethyl)aminomethane hydrochloride (Sigma-Aldrich) and 25 mM EDTA, resuspended by vortexing, and transferred to 2-ml Lysing Matrix E tubes (MP Biomedicals). The cells were disrupted for two min in a Mini-BeadBeater-24 (BioSpec Products) and centrifuged at 16,000 RCF for 90 s. Following bead beating, DNA was further extracted and purified according to an enzymatic protocol as described in Supplemental Methods 2 in Text S1. DNA was quantified using Quanti-iT PicoGreen double-stranded DNA (dsDNA) assay kit (Thermo Fisher) and Spectramax Gemini XS microplate spectrofluorometer (Molecular Devices).

### Quantitative PCR.

qPCR was used to quantify the relative abundance of each member of the biofilm community by targeting the single-copy gene, *rpoC*, as a proxy for counting the genomes of each organism. A set of unique primers and probes were designed against each the *rpoC* gene of each species (see Table S1 in the supplemental material). The genomic sequences used to design the primers and probes were the assembled genomes of isolated organisms (strains HL-48, HL-49, HL-53, HL-55, HL-58, HL-91, HL-93, HL-109, and HL-111) or the reconstructed genomes for species not yet isolated (bins 01, 04, 11, 16, and 18; Supplemental Methods 3 in Text S1). Primers and probes were ordered from Integrated DNA Technologies (Coralville, IA). Probes included 5′ 6-carboxyfluorescein (6-FAM) reporter, internal ZEN quencher, and 3′ Iowa-Black FQ quencher. A standard curve in triplicate was included for each target on every plate, consisting of 3 × 10^6^, 3 × 10^5^, 3 × 10^4^, 3 × 10^3^, 3 × 10^2^, and 3 × 10^1^ copies. The standard curves were generated using DNA extracted from isolates or plasmids with the *rpoC* gene cloned for organisms not isolated. All samples were assayed in triplicate and included three no-template controls for each target. The TaqMan Fast Universal PCR Master Mix (Thermo Fisher) was used at a total volume of 20 μl per well with 0.50 μM concentration for each primer, 0.25 μM concentration of the probe, and 1 ng of genomic DNA (gDNA). qPCR was performed using the Step One Plus real-time PCR system (Thermo Fisher), beginning with 95.0°C for 20 s, followed by 40 cycles, with 1 cycle consisting of 95.0°C for 2 s and 62.0°C for 30 s.

### Stable isotope tracers for quantifying C and N uptake into bulk biomass.

Two double-isotope labeling treatments were performed on 7-day-old UCC-O cultures to assess C and N incorporation: Na^15^NO_3_ and NaH^13^CO_3_ (unamended) and ^15^NH_4_Cl and NaH^13^CO_3_ (amended). Unamended, heavy-isotope-enriched medium was prepared by adding 4.4 mM Na^15^NO_3,_ 13.2 mM NaNO_3,_ 5 mM NaH^13^CO_3,_ and 5 mM NaHCO_3_ to nitrogen-and-carbon-free (NC-free) HLA-400 medium. Amended, heavy-isotope-enriched medium was prepared by adding 17.6 mM NaNO_3,_ 1.25 mM ^15^NH_4_Cl, 3.75 mM NH_4_Cl, 5 mM NaH^13^CO_3_, and 5 mM NaHCO_3_ to NC-free HLA-400 medium. For each labeling experiment, cultivation medium was aspirated from 25-ml tissue culture flasks (*n* = 3) that received 10 ml of the respective labeled medium and incubated for 2, 8, or 16 h. Following incubation, the labeled medium was removed, and the biofilms were washed three times with 10 ml NC-free HLA-400 medium to remove any residual isotope label. Biofilm pellets were collected by scraping the culture flasks with 5 ml of NC-free HLA-400 medium, followed by centrifugation at 21,000 RCF for 10 min at 4°C. The pellets were stored at –80°C until analysis. Natural abundance samples for each treatment (*n* = 3) received 10 ml of the respective medium without the isotope label and were collected as described above. In order to account for nonbiological labeling, wash controls for each treatment (*n* = 3) were performed by incubating biofilms with 10 ml of labeled medium for 1 min before being washed as described above.

### Isotope ratio mass spectrometry (IRMS).

C and N stable isotope content was analyzed using a Costech Analytical (Valencia, CA) elemental analyzer (EA) (ECS 4010 CHNSO analyzer) coupled to a Thermo Scientific Delta V Plus isotope ratio mass spectrometer. In preparation for isotope analysis, we lyophilized the mat samples and then homogenized them using a mortar and pestle. Sample aliquots were weighed in tin capsules for elemental analysis, the combustion reactor (with cobaltic oxide and chromium oxide catalyst) was maintained at 1,020°C, and the reduction reactor (with copper catalyst) was maintained at 650°C. In-house glutamic acid isotope standards were calibrated against USGS 40 and USGS 41 (δ^13^C of −26.39‰ and +37.63‰ respectively). Due to the large isotopic enrichment observed in some samples, we used a series of in-house standards, introduced following analysis of a labeled sample, to ensure no sample carryover between samples. A two-point correction was performed using only the standard values that were void of carryover. We report all isotope content in standard delta (δ) notation:

(1) $$\text{δ}=\left(\frac{R_{\text{sample}}}{R_{\text{standard}}}-1\right)\times 1,000\text{‰}$$

where *R*_sample_ is the respective measured ^13^C/^12^C or ^15^N/^14^N ratio of a sample and *R*_standard_ is the ratio of a standard sample. In this case, we reference all ^13^C isotope measurements to Vienna Pee Dee Belmnite (VPDB; *R*_standard_ = 0.0112372) and all ^15^N isotopic measurements to atmospheric N_2_ (AIR) (*R*_standard_ = 0.003676).

### Protein extraction, digestion, and HPLC-MS/MS analysis.

Biofilm cell pellets (single biological replicates) were suspended in 100 mM ammonium bicarbonate buffer (pH 8.0) and subjected to bead beating in Bullet Blender homogenizer (Next Advance Inc.) for 3 min with 0.1-mm zirconia/silica beads (Biospec Products, Inc.). Following cell lysis, global protein fractions were extracted from the cell lysates using established protocols and were analyzed using high-performance liquid chromatography coupled to tandem mass spectrometry (HPLC-MS/MS) (35) (Supplemental Methods 4 in Text S1).

### Metaproteomic data analysis.

For the global proteomic analysis, MS/MS spectra were searched against a protein collection assembled from draft genomes of isolates from UCC-O and reconstructed genomes of unisolated community members (15), as well as the contaminant sequences for bovine trypsin, human keratin, and serum albumin precursor. The MS-GF+ search algorithm was used to match MS/MS spectra to peptide sequences (36). Partially tryptic cleavage, dynamic modification of methionine oxidations, and maximum 20 ppm parent ion mass tolerance were included in the search. Peptide identifications were retained at a <1% false-discovery rate (FDR) (Table S3). Protein function predictions were previously performed as described by Lindemann et al. (10). Potential shifts in organism relative abundance due to treatment condition were accounted for by dividing a sample’s peptide counts for a protein by the total peptide counts for the organism from which that protein originated. This approach normalized differences in protein expression across total proteins observed for a species, which is an analog for a species’ overall contribution to community protein content.

### Stable isotope probing for high-lateral-resolution secondary ion mass spectrometry (NanoSIMS).

Two double-isotope labeling treatments were performed on 7-day UCC-O cultures to assess carbon and nitrogen incorporation (Na^15^NO_3_ and NaH^13^CO_3_, unamended; and ^15^NH_4_Cl and NaH^13^CO_3,_ amended). In order to account for any potential effects of stable isotope labels on uptake in the cultures, four single-isotope labeling treatments served as controls (Na^15^NO_3_ and NaHCO_3_; ^15^NH_4_Cl and NaHCO_3_; NaNO_3_ and NaH^13^CO_3_; NH_4_Cl and NaH^13^CO_3_). Prior to label addition, natural abundance wafers (*n* = 3) were collected from each tissue culture dish and fixed with 4% paraformaldehyde (prepared in NC-free HLA) in a sterile tissue culture dish. Time course isotope labeling experiments were conducted for 2, 8, and 16 h, with wafers (*n* = 3) collected at each time point. The biofilms on the silicon wafers were also fixed with 4% paraformaldehyde and stored at 4°C for at least 24 h. Prior to nanoscale secondary ion mass spectrometry (NanoSIMS) analysis, the silicon wafers were dehydrated with ethanol as described previously (37).

### NanoSIMS analysis.

SIMS was performed using a Cameca NanoSIMS 50L microprobe (Gennevilliers, France) housed in the Environmental Molecular Science Laboratory. Prior to analysis, samples were coated with 10 nm of Au to minimize charging during analysis (19, 38). High-current sputtering was performed with the Cs^+^ primary ion beam prior to collecting data, where samples were dosed with ∼2 × 10^16^ ions/cm^2^ (a depth of ∼100 nm) to achieve sputtering equilibrium (19). An ∼1.5-pA Cs^+^ primary ion beam was used for all analyses, where the ^16^O^−^, ^12^C^12^C^−^, ^12^C^13^C^−^, ^12^C^14^N^−^, ^12^C^15^N^−^, and ^31^P^−^ secondary ions were detected simultaneously. For standard analysis, 40 μm by 40 μm images were acquired at 256 pixels by 256 pixels and 2 ms/pixel over 12 to 15 planes. For the high-throughput analyses used for image statistics, 30 μm by 30 μm images were acquired at 128 pixels by 128 pixels and 3 ms/pixel over 10 planes.

### NanoSIMS image processing.

NanoSIMS image visualization was performed using OpenMIMS (a free ImageJ plugin courtesy of the National Resource for Imaging Mass Spectrometry). Images were summed over all planes. Images opened and processed within OpenMIMS were then analyzed using a custom-written Python module and scripts (available upon request). Python (v2.7.10) was implemented using WinPython (v2.7.10.1, http://winpython.github.io), a free, open-source, and portable full-featured Python-based scientific environment. The NanoSIMS analysis module depends upon the following packages: Numpy (39) (v1.9.3), Scipy (39) (v0.16.1), Scikit-Image (40) (v0.11.3), Matplotlib (41) (v1.5.0), and Colorspacious (v1.1.0, https://pypi.python.org/pypi/colorspacious). IPython (42) (v3.2.0), an enhanced Python shell, was used within the Scientific Python Development Environment (Spyder v2.3.5.2) for interactively creating figures and cell geometry libraries. C and N enrichment were first calculated using equation 1. Background was then removed from images using manual thresholding as well as the Scikit-Image module. For images, the data were normalized, and values in the bottom 5% and upper 95% were set to the 5th and 95th percentile value, respectively, in order to remove noise. Background pixels were colored black, and all other pixels were colored according to their value and where it fell on the cividis (43) colormap. Then, pixels within 2 pixels of the closest background pixel were faded toward black by altering their lightness (*J*’) value in CIECAM02-UCS colorspace (44) using the Colorspacious module. For histograms and biplots, regions high in phosphate were removed. Autotroph and heterotroph pixels were separated out using a method similar to that reported previously (20). Heterotrophs on top of the cyanobacterial cells were not included in the analysis, but heterotrophic cells attached on the side of the cyanobacterial filaments were included. Calculation of the Wilcoxon signed rank test was performed using SciPy.
