# Supplementary material for: Nitrogen Source Governs Community Carbon Metabolism in a Model Hypersaline Benthic Phototrophic Biofilm
Source: mSystems. 2020 Jun 9;5(3):e00260-20. doi: 10.1128/mSystems.00260-20 (PMC7289588; doi:10.1128/mSystems.00260-20)
Supplement: TABLE S1 [file mSystems.00260-20-st001.docx]

**Table S1.**

| Metagenomic bin | Taxonomy | Primer/Probe | Sequence (5’-3’)^a^ |
| --- | --- | --- | --- |
| 01 | Bacteroidetes | Forward  Probe  Reverse | CTGAGTCGATGCACAAGTCTAA  ACCTGCGTTATGTTGAGTATGATGATGGTG  CACGGCTGAGTACTACGTTATG |
| 02 | HL-53 | Forward  Probe  Reverse | AGTGTGGTCGTGCTTGAAA  AGCCGTGAAACTTGTAGACGCGAA  CCGGCAATGTTTACGTCTTTAC |
| 03 | HL-58 | Forward  Probe  Reverse | TCGGTCAAGCATGGTGATTC  ATGATCGGGTGAGTGTGCGGATC  CAGTGATGCCCTGATCCATATT |
| 04 | HL-87 | Forward  Probe  Reverse | TTGCCTCGAAGGTGGTTATC  AGCTGGATTTCGATGACGTACCGC  CCCACCGACAGCATGTAAT |
| 05 | HL-91 | Forward  Probe  Reverse | CTCCGGGACGATCGAATTG  CGCAATGCCCACAGTCTGCAAA  GGCATCGATGATGACGATCT |
| 06 | HL-93 | Forward  Probe  Reverse | GCTCCTCTGCGGTTGATAG  AAGTGAAGCATGGCGGCAAAGTG  GCGCACTCGAACGAGATA |
| 10 | HL-49 | Forward  Probe  Reverse | GACCACCAATAAGGATGGAGAG  ATGGGACGATCTGGTGAGATTCGA  GCTCCGTAAGGAACGTGATT |
| 11 | ANA | Forward  Probe  Reverse | GCGGTCTTGCACATTGATAAC TGGTCTGGTGCTGCTGGTATTTGA  ATACGCGGTAAACCCTGAATAA |
| 13 | HL-48 | Forward  Probe  Reverse | GACAGCGTCCAGGTCAAG  CTACGTGCTTGATGTTGTGCAGGC  TCGAGAGACCACTACCAGTT |
| 14 | HL-55 | Forward  Probe  Reverse | GCGTCTGCAGTGGACAATA  ACGCCTGCATAACCTGAAGTCCAT  GAGACACAACCACCAGAGAAC |
| 15 | HL-111 | Forward  Probe  Reverse | GACGGTCGCATCGAATACC  ATGCCCACGATCACCGACAAGAAA  TAGGGCACCTTGTGCATTT |
| 16 | OSCR | Forward  Probe  Reverse | GGAAATTGCCTGTCGCAATG  ATTCGTGAAGGGACTGAAGCCACT  GCTAAAGCGATCCTCCTTACTG |
| 17 | HL-109 | Forward  Probe  Reverse | GCTGTTCATCGCATCCAGTA  CTGCGTGTCGATGAGGGTGACA  AGGATCGGGCGGGAATA |
| 18 | Rhodo | Forward  Probe  Reverse | AAGTGAATGTCGAGGGCAAG  AACCTGCTGAAGAACGCCGC  GGTGATGACCAGTGTCATGTT |
| 21 | HL-46 | Forward  Probe  Reverse | AGATGCACAAGGTGCCTTAC  TACGGTGCTGATGTTCAAGGACGG  CCCGATTGTTCGGTGATGAT |

^a^All probes were modified with 5’ 6-FAM and 3’ lowa Black FQ in addition to the internal ZEN modification.
