# Supplementary material for: Nitrogen Source Governs Community Carbon Metabolism in a Model Hypersaline Benthic Phototrophic Biofilm
Source: mSystems. 2020 Jun 9;5(3):e00260-20. doi: 10.1128/mSystems.00260-20 (PMC7289588; doi:10.1128/mSystems.00260-20)
Supplement: TABLE S2 [file mSystems.00260-20-st002.docx]

**Table S2.**

| **Locus tag** | **Annotation** | **Peptide counts** | |
| --- | --- | --- | --- |
|  |  | **NO3- only** | **NO3- +NH4+** |
| Ga0058931_0607 | Respiratory nitrate reductase beta subunit (NarH) [EC:1.7.99.4] | 28 | 9 |
| Ga0058931_0606 | Respiratory nitrate reductase, alpha subunit (NarG) [EC:1.7.99.4] | 71 | 37 |
| Ga0058931_2059 | Nitrogen regulation protein (NtrC) | 16 | 1 |
| Ga0058931_3035 | Nitrogen regulatory protein P-II 1 (GlnB) | 48 | 0 |
| Ga0058931_2930 | Nitrogen regulatory protein P-II 1 (GlnB) | 26 | 7 |
| Ga0058931_0932 | Nitrogen fixation protein of unknown function | 14 | 3 |
| Ga0058931_1220 | Nitrogen-fixing NifU domain protein | 25 | 10 |
