# Supplementary figures and images for: Nitrogen Source Governs Community Carbon Metabolism in a Model Hypersaline Benthic Phototrophic Biofilm
Source: mSystems. 2020 Jun 9;5(3):e00260-20. doi: 10.1128/mSystems.00260-20 (PMC7289588; doi:10.1128/mSystems.00260-20)

**
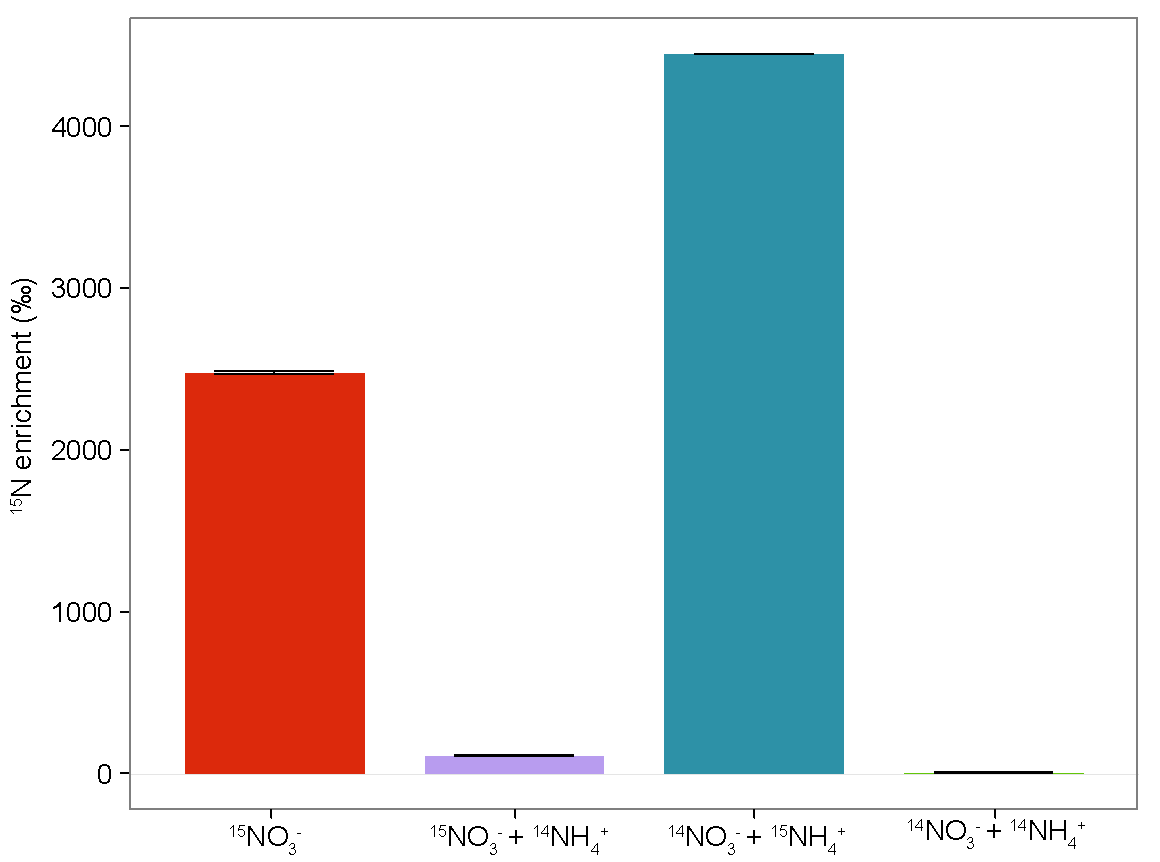
**

**Fig. S1**

Supplement: FIG S1 [file mSystems.00260-20-sf001.docx]

**
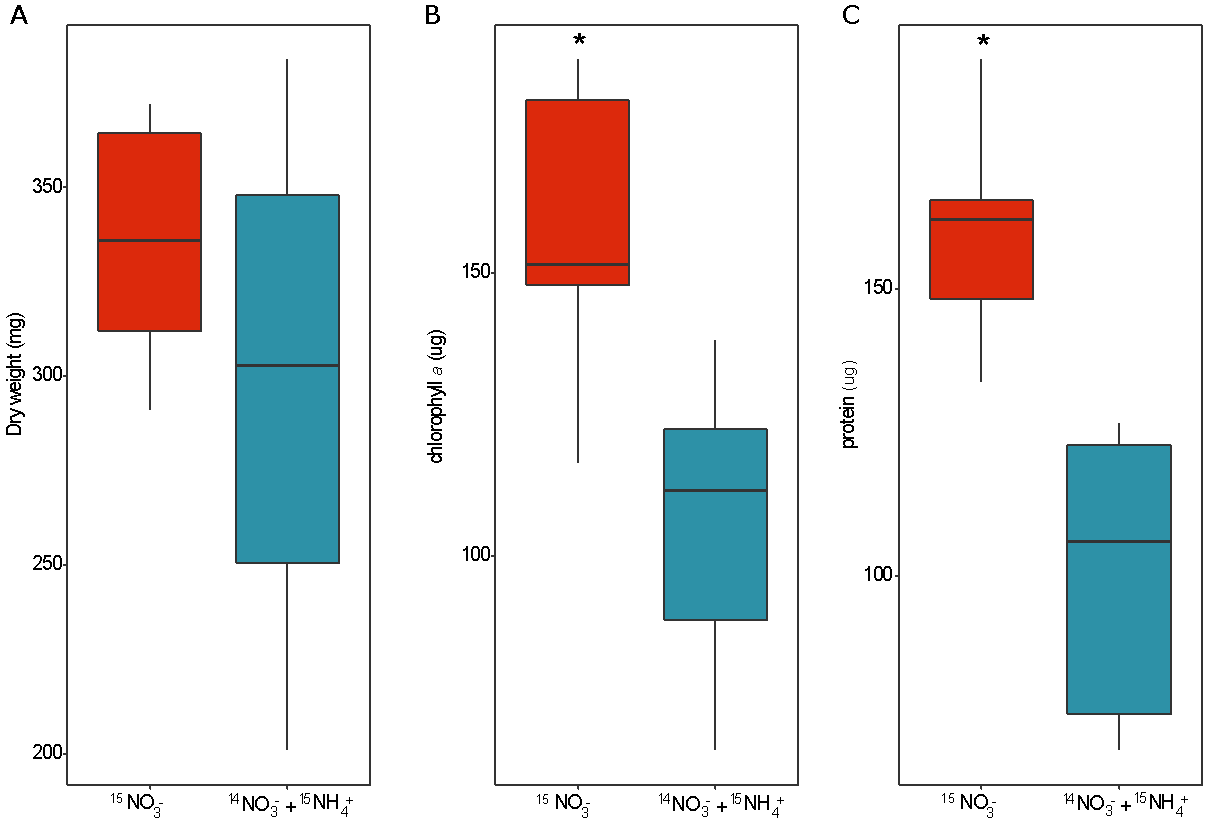
**

**Fig. S2**

Supplement: FIG S2 [file mSystems.00260-20-sf002.docx]

**
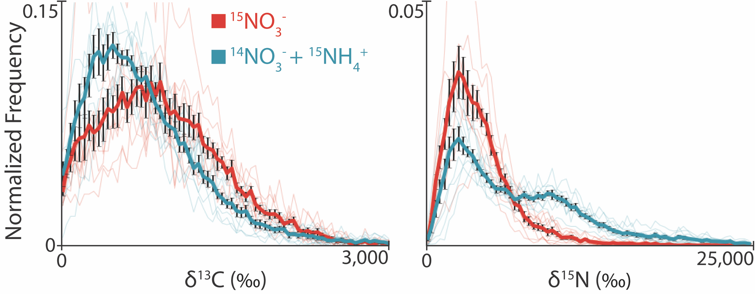
**


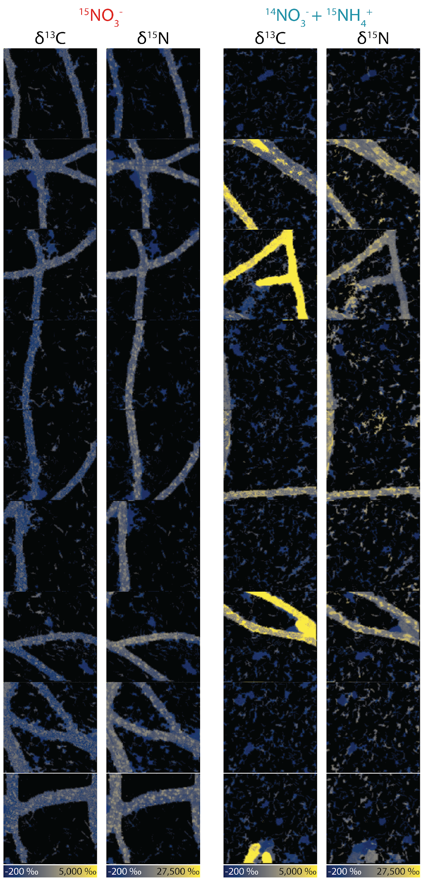


**Fig. S3**

Supplement: FIG S3 [file mSystems.00260-20-sf003.docx]

**
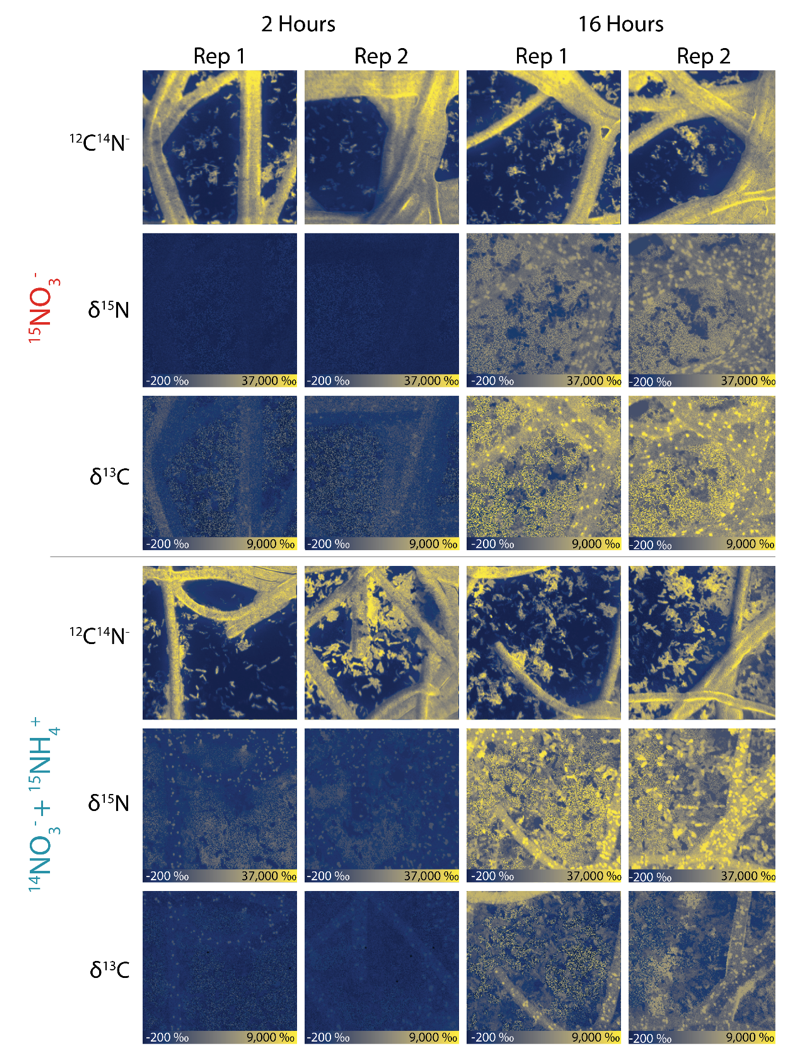
**

**Fig. S4**

Supplement: FIG S4 [file mSystems.00260-20-sf004.docx]

**
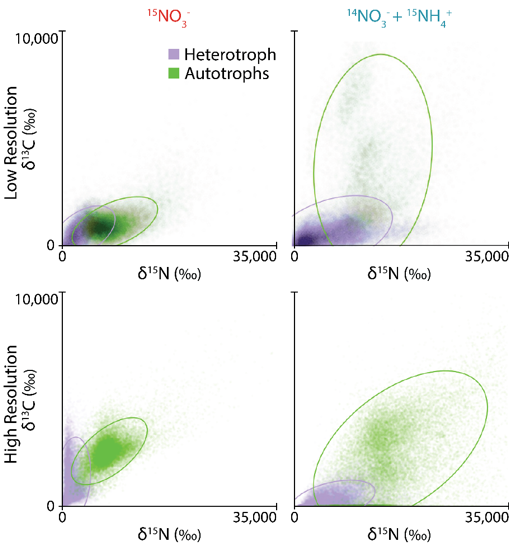

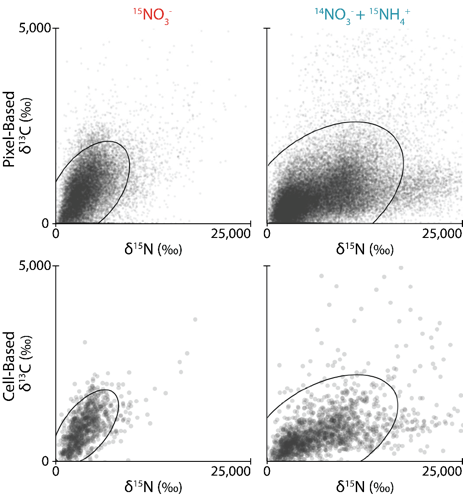
**

**Fig. S5**

Supplement: FIG S5 [file mSystems.00260-20-sf005.docx]

**
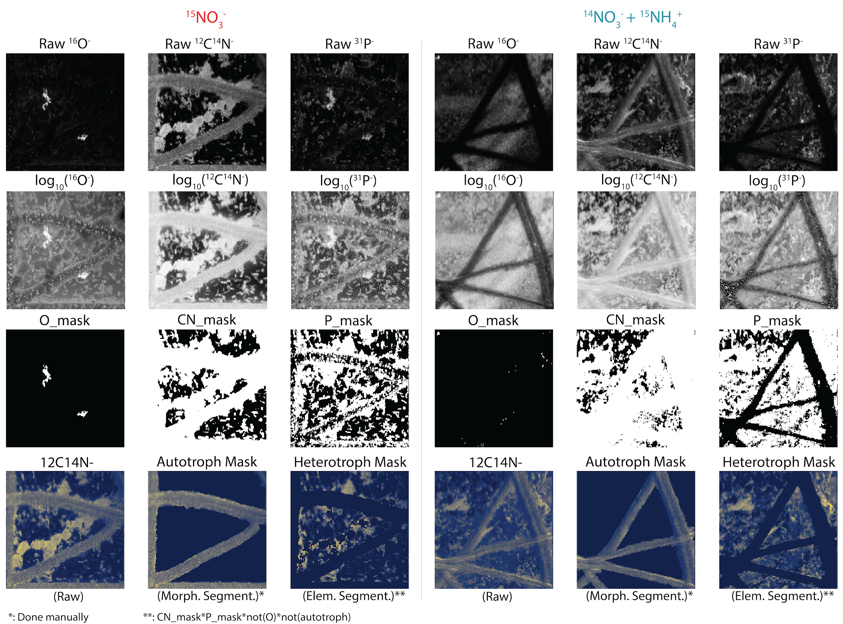
**

**Fig. S6**

Supplement: FIG S6 [file mSystems.00260-20-sf006.docx]
