# Supplementary material for: Nitrogen Source Governs Community Carbon Metabolism in a Model Hypersaline Benthic Phototrophic Biofilm
Source: mSystems. 2020 Jun 9;5(3):e00260-20. doi: 10.1128/mSystems.00260-20 (PMC7289588; doi:10.1128/mSystems.00260-20)
Supplement: TEXT S1 [file mSystems.00260-20-s0001.docx]

**Nitrogen source governs community carbon metabolism in a model hypersaline benthic phototrophic biofilm**

Christopher R. Anderton^1^*, Jennifer M. Mobberley^2,3^, Jessica K. Cole^2^, Jamie R. Nunez^2^, Robert Starke^1^, Amy A. Boaro^2^, Yasemin Yesiltepe^2^, Beau R. Morton^2^, Alexandra B. Cory^2^, Hayley C. Cardamone^2^, Kirsten S. Hofmockel^1^, Mary S. Lipton^1^, James J. Moran^1^, Ryan S. Renslow^2,4^, James K. Fredrickson^2^, Stephen R. Lindemann^2,5,6^*

^1^Environmental Molecular Sciences Laboratory, ^2^Biological Sciences Division, ^3^National Security Directorate, Pacific Northwest National Laboratory, 902 Battelle Boulevard, Richland, WA USA 99354; ^4^Gene and Linda Voiland School of Chemical Engineering and Bioengineering, Washington State University, Pullman, WA USA 99164; ^5^Whistler Center for Carbohydrate Research, Department of Food Science, Purdue University, West Lafayette, IN, United States, ^6^Department of Nutrition Science, Purdue University, West Lafayette, IN, United States

[*Christopher.Anderton@pnnl.gov](mailto:*Christopher.Anderton@pnnl.gov); 902 Battelle Boulevard, Richland, Washington 99354; 509-371-7970

[*lindemann@purdue.edu](mailto:*lindemann@purdue.edu); 745 Agriculture Mall Drive, West Lafayette, Indiana 47907; 765-494-9207

**This document includes:**

**Supplemental Methods**

**Supplemental Methods 1.** Biofilm biomass characterization

**Supplemental Methods 2.** DNA extraction

**Supplemental Methods 3.** Clone library construction for reconstructed genomes.

**Supplemental Methods 4.** Protein extraction, digestion, and HPLC-MS analysis

**Supplemental Methods 1. Biofilm biomass characterization**

***Dry weight measurements.*** Three biological replicates were quantified per culture, where the frozen one mL aliquoted homogenized biofilm pellets were transferred to preweighed, two-mL microcentrifuge tubes and incubated at 45 ˚C until dry, indicated by the weights of the pellets ceasing to decrease. The final weight was recorded, and total dry weight was calculated by subtracting the original mass of the empty tube from the final weight of the tube and dried biomass.

***Chlorophyll quantification.*** Three biological replicates, each divided into three technical replicates of the frozen one mL aliquoted homogenized biofilm pellets, were quantified per culture. The frozen pellets were thawed on ice, resuspended in one mL of pure methanol, and incubated in the dark at 4 °C for 1 hr. Insoluble biomass was pelleted by centrifugation at 15,000 RCF at 4 °C for 5 min. Absorption of the supernatant was measured at 655 nm using a SmartSpec Plus spectrophotometer (BioRad). To calculate chlorophyll α concentration (µg/mL), a standard curve was constructed using absorbance of spinach-derived chlorophyll *a* (Sigma-Aldrich) in pure methanol, with concentrations ranging from 2 to100 µg/mL.

***Total protein analysis.*** Three biological replicates, each divided into three technical replicates of the frozen one mL aliquoted homogenized biofilm pellets, were quantified per culture. Culture pellets were thawed on ice and resuspended in 300 µL of B-PER Reagent (Pierce, Thermo Fisher) to extract total protein and peptides. The assay was performed according to the manufacturer’s instructions for the microplate procedure. Absorption was measured at 562 nm using a SpectraMax Plus 384 microplate reader (Molecular Devices). Protein concentration was calculated using a standard curve, consisting of 12 points ranging from 100 to 1,200 µg/mL, prepared using bovine serum albumin from the BCA kit.

**___________________**

**Supplemental Methods 2. DNA extraction**

Supernatants from bead-beating were transferred to two-mL microcentrifuge tubes, incubated at 85 °C for five min, and cooled to room temperature on the bench top. Then, 70 µL of 10 % SDS (Sigma-Aldrich) was added and tubes were vortexed until the contents were thoroughly mixed. Proteinase K (New England BioLabs) was added to a final concentration of 0.2 mg/mL and tubes were incubated at 56 °C for one hour with shaking at 1,000 RPM. 100 µL of 5 M NaCl was added to each sample followed by 100 µL of a 65 °C solution of hexadecyltrimethylammonium bromide (0.274 M; Sigma-Aldrich) and NaCl (0.702 M), tubes were vortexed until thoroughly mixed, and then incubated at 65 °C for 10 min. Each tube was extracted by the addition of one volume of chloroform-isoamyl alcohol (24:1; Sigma-Aldrich), vortexed for 1 min, and centrifuged at 16,000 RCF at 23 °C at 10 min. The aqueous phases were transferred to new microcentrifuge tubes and the extraction repeated with phenol-chloroform–isoamyl alcohol mixture (25:24:1; Sigma-Aldrich), followed by an extraction using chloroform/isoamyl alcohol (24:1; Sigma-Aldrich). 10 µg of RNase A (Thermo Fisher) were added to each tube and they were incubated at 37 °C for 30 min. One-tenth of a volume of sodium acetate (3 M, pH 5.5; manufacturer) and 2.5 volumes of ice-cold ethanol (200 proof; Decon Laboratories, King of Prussia, PA, USA) were added, tubes were inverted for one min, and incubated at -80 °C for 30 min. They were then centrifuged at 16,000 RCF at 4 °C for 10 min and the supernatants were removed. The DNA pellets were washed twice by adding 0.5 mL of ice-cold ethanol (70%) and centrifuged at 16,000 RCF at 4 °C for 10 min. The pellets were dried in a Savant SC100 SpeedVac Concentrator (Thermo Fisher) on medium heat until no ethanol remained and then resuspended in 200 µL of Tris-EDTA buffer solution (pH 7.4, Fluka).

**___________________**

**Supplemental Methods 3. Clone library construction for reconstructed genomes.**

Primers flanking the *rpoC* genes for those organisms not in isolation (bins 01, 04, 11, 16, and 18) were designed for each metagenomic bin(1). The *rpoC* genes were cloned for use as standards during qPCR. Polymerase chain reaction was performed with the Phusion High-Fidelity DNA Polymerase (New England BioLabs) per manufacturer instructions. The amplified products were cloned into the pCR 4-TOPO plasmid vector using the Zero Blunt TOPO PCR Cloning Kit (Thermo Fisher). Plasmids were extracted using the QIAprep Spin MiniPrep Kit (Qiagen, Valencia) and linearized by digestion with restriction enzymes SpeI or XbaI (New England BioLabs) per manufacturer instructions prior to use as standards.

**___________________**

**Supplemental Methods 4. Protein extraction, digestion, and HPLC separation analysis**

Proteins were denatured and reduced under following conditions: 8M Urea, 5 mM DTT, 100 mM ammonium bicarbonate buffer at 60 °C for 45 min with vigorous (1000 rpm) shaking in Thermomixer R (Eppendorf). After denaturation, samples were diluted 8-fold with 100 mM ammonium bicarbonate and calcium chloride was added to achieve a concentration of 1mM. Tryptic digestion was performed for 3 hours at 37˚C with 1:50 (w/w) trypsin to protein ratio. The digested sample was desalted and cleaned via solid phase extraction (SPE) C18 (Supelco). Sample was concentrated in Speed-Vac (Thermo Savant) before performing BCA Assay to determine final peptide concentration.

Peptide samples were subsequently separated at a flow rate of 0.5 ml/min on a reversed phase Waters XBridge C18 column (250 mm × 4.6 mm column containing 5 μm particles, and a 4.6 mm × 20 mm guard column) using an Agilent 1200 HPLC System equipped with a quaternary pump, degasser, diode array detector, Peltier-cooled autosampler and fraction collector (both set at 4 oC). Approximately 400 µg of tryptic peptides were suspended in buffer A (10 mM ammonium formate, pH 10.0) and loaded onto the column. The LC gradient started with a linear increase of solvent A to 10% B (10 mM ammonium formate, pH 10.0, 90% acetonitrile) for 3 min, then linearly increased at 86 min to 30% B, 10 min to 42.5% B, 5 min to 55% B, and another 5 min to 100% solvent B. Using an automated fraction collector, 96 fractions from 11 min through 122 min of the gradient were collected for each sample, lyophilized and reconstituted into 24 fractions prior to LC-MS/MS analysis.

A custom Waters nano-Acquity dual pumping UPLC system was configured for on-line trapping. The injection volume was 5µL at 3µL/min with reverse direction elution onto the analytical column at 300nL/min. Fused silica columns (360µm o.d.) were packed in house (Polymicro Technologies Inc.) with media retention by way of 1-cm sol-gel frits (2). Jupiter C18 media (Phenomenex) was used in both the trapping column (5 µm particles, 150 µm i.d. x 4 cm long) and analytical column (3 µm particles, 75 µm i.d. x 70 cm long). Mobile phase A consisted of 0.1% formic acid in water, while mobile phase B was 0.1% formic acid in acetonitrile. The following gradient profile was used (min, %B): 0, 1; 2, 8; 20, 12; 75, 30; 97, 45; 100, 95; 110, 95; 115, 1; 150, 1. A Q-Exactive Plus mass spectrometer was used for sample analysis (Thermo Scientific). A chemically etched fused silica electrospray emitter made on-site (150 µm o.d. x 20 µm i.d.)(3) was mounted on a homemade bracket at the MS source inlet and held at 2.2 kV, while the source ion transfer tube temperature was maintained at 350 ^o^C. Precursor spectra were acquired for 100 min from 400-2000 m/z at 35k mass resolution with data dependent acquisition of product spectra of the most intense ions (top 12) at a resolution of 17.5k using a normalized collision energy of 30.

**References:**

1. Nelson WC, Maezato Y, Wu Y-W, Romine MF, Lindemann SR. Identification and Resolution of Microdiversity through Metagenomic Sequencing of Parallel Consortia. Applied and Environmental Microbiology. 2016;82(1):255-67.

2. Maiolica A, Borsotti D, Rappsilber J. Self-made frits for nanoscale columns in proteomics. PROTEOMICS. 2005;5(15):3847-50.

3. Kelly RT, Page JS, Luo Q, Moore RJ, Orton DJ, Tang K, et al. Chemically Etched Open Tubular and Monolithic Emitters for Nanoelectrospray Ionization Mass Spectrometry. Analytical Chemistry. 2006;78(22):7796-801.
